# Clinical Evaluation of on-Table Extubation in Patients Aged Over 60 Years Undergoing Minimally Invasive Mitral or Aortic Valve Replacement Surgery

**DOI:** 10.3389/fsurg.2022.934044

**Published:** 2022-06-29

**Authors:** Yunfen Ge, Yue Chen, Zhibin Hu, Hui Mao, Qiong Xu, Qing Wu

**Affiliations:** ^1^ Center for Rehabilitation Medicine, Department of Anesthesiology, Zhejiang Provincial People’s Hospital (Affiliated People’s Hospital, Hangzhou Medical College), Hangzhou, China; ^2^ Heart Center, Department of Cardiovascular Surgery, Zhejiang Provincial People’s Hospital (Affiliated People’s Hospital, Hangzhou Medical College), Hangzhou, China; ^3^ Center for Reproductive Medicine, Department of Gynecology, Zhejiang Provincial People’s Hospital (Affiliated People’s Hospital, Hangzhou Medical College), Hangzhou, China

**Keywords:** mitral or aortic valve disease, on-table extubation, delayed extubation, aortic occlusion clamping, cardiopulmonary bypass time

## Abstract

**Aims:**

To evaluate the clinical efficiency of on-table extubation (OTE) versus delayed extubation in patients aged over 60 years that underwent minimally invasive mitral or aortic valve replacement surgery and evaluate the factors associated with successful OTE implementation.

**Materials:**

Patients over 60 years with mitral or aortic valve disease who received minimally invasive mitral or aortic valve replacement surgery from October 2020 to October 2021 were selected retrospectively. We divided patients into the on-table extubated (OTE) group (*n* = 71) and the delayed extubation (DE) group (*n* = 22). Preoperative, intraoperative, and postoperative clinical variables were compared between the two groups.

**Results:**

Patients in the DE group underwent longer surgery time, longer aortic occlusion clamping time and longer cardiopulmonary bypass time than those in the OTE group(217.48 ± 27.83 vs 275.91 ± 77.22, *p* = 0.002; 76.49 ± 16.00 vs 126.55 ± 54.85, *p* = 0.001; 112.87 ± 18.91 vs 160.77 ± 52.17, *p* = 0.001). Patients in the OTE group had shorter postoperative mechanical ventilation time (min), shorter ICU time, shorter postoperative hospital length of stay and lower total cost and medication cost (*p* < 0.05). The AUC for aortic occlusion clamping time was 0.81 (*p* < 0.01), making it the most significant predictor of on-table extubation success.

**Conclusions:**

On-table extubation following mitral or aortic valve cardiac surgery was associated with a superior clinical outcome and high cost-effectiveness.

## Introduction

Postoperative ventilation was traditionally thought to be required for maintaining patient stability following cardiac surgery; however, long-term mechanical ventilation may raise the risk of unfavorable outcomes in the postoperative period, particularly in elderly patients. A recent study showed that a prolonged duration of mechanical ventilation (DMV) decreases mid-term survival rates after coronary artery bypass grafting (CABG) (1). Fast-track cardiac anesthesia (FTCA) provides an alternative to standard cardiac surgical care by improving efficiency without compromising safety or clinical results (2). FTCA may result in more efficient use of resources, especially if intensive care unit (ICU) beds are scarce (3). Meanwhile, the benefits of early extubation were recognized in cardiac surgery and supported by several previous studies (4).

In our current practice, on-table extubation (OTE) was attempted even for patients who underwent complicated cardiac surgery. The factors that preclude OTE are bleeding, hypothermia, cardiovascular instability, post-operation pain, post-cardiopulmonary bypass (CPB) pulmonary injury leading to reintubation and an increase in morbidity (5, 6). This study was undertaken to evaluate the efficacy of on-table extubation versus delayed extubation in terms of postoperative complications, length of stay (LOS) in ICU, patient recovery, and hospital LOS in patients aged over 60 years that received minimally invasive mitral or aortic valve replacement surgery and to evaluate the factors associated with successful implementation of OTE.

## Methods

### Patients and Methods

It is a single-centre, retrospective observational study. A total of 93 patients aged over 60 years with mitral or aortic valve disease were selected from October 2020 to October 2021 in Zhejiang Provincial People’s Hospital, China. This study was approved by the ethics committees of this hospital (No. 2018KY034). Individual consent was waived owing to the retrospective nature of the study. All interventions were performed by the same surgical team.

The inclusion criteria were as follows: (1) patients above 60 years of age; (2) patients presenting with isolated mitral or aortic valve lesions; (3) Eurscore ≤5 (7). The exclusion criteria were as follows: (1) patients with potential surgical complications, such as severe chest deformity and a history of right chest surgery; (2) patients with femoral arteriovenous malformations; (3) patients with severe valvular heart disease whose cardiac structure form had seriously changed with left ventricular end-diastolic diameter >60 mm or ejection fraction (EF) <50%; (4) patients in poor general condition, accompanied by multiple organ failure, such as patients with New York Heart Association (NYHA) class grade IV heart failure (7), showing no improvement after treatment; (6) patients having emergency surgery, redo-surgery; (7) moderate pulmonary hypertension (pulmonary artery systolic pressure ≥50 mmHg); (8) patients with chronic obstructive pulmonary diseases.

### Anesthesia

Anesthesia was administered by the same team during the perioperative period. Our department’s protocol for anesthesia was as follows: etomidate 0.2 mg/kg, propofol (1–2 mg/kg), sufentanil (0.5–1 µg/kg) and rocuronium bromide (0.6 mg/kg) were used to induce anesthesia. Continuous venous infusion of remifentanil (0.2 µg/kg/min), rocuronium bromide (7 µg/kg/min) and propofol (3–8 mg/kg/h) was used for the maintenance of anesthesia during surgery. Before chest closure, sufentanil (40–60 µg) was administered. The bispectral index (BIS) was utilized as an indicator of anesthesia level (depth) and was kept between 40 and 60. After induction, a single-lumen endotracheal tube was used to intubate the patient in the supine position, with the patient’s arm abducted at an angle of 90°. The blocks were performed as originally described by Blanco and colleagues (8), serratus anterior plane block (SAPB) was performed with a 22-gauge, 50-mm needle (Pajunk Medical Systems, Jiangsu, China) under ultrasound in-plane guidance. The target site was the fascial plane between the serratus anterior muscle posterior surface and lateral periosteum of the fifth right rib on the midaxillary line; ropivacaine 0.375% 150 mg was injected into the serratus anterior muscle under continuous ultrasound guidance. Then the patient was placed in the supine position and tilted (30°) to the left with a slight elevation of the right side using a pad. Transoesophageal echocardiography was used during the operation. All of the TTE was performed by 2 sonographers with at least 20 years of experience. Rocuronium bromide was stopped when the primary intervention was over.

### Thoracoscopy-Assisted Minimally Invasive Mitral or Aortic Valve Replacement

A 3-cm longitudinal incision was made in the right groin area to expose the femoral artery and vein (incision 1) for cardiopulmonary bypass (CPB). Additionally, a 4–6 cm curved incision was made along the lower margin of the right breast to gain access to the thoracic cavity *via* the fourth intercostal space (incision 2). A rib spreader was used to expand the intercostal space. The pericardium was incised, and the aortic root was exposed. Systemic heparinization was achieved, and the femoral artery was cannulated using a femoral artery cannula, and a vena cava cannula was cannulated from the femoral vein to the superior vena cava, respectively. The cardioplegia cannula was inserted into the aortic root following the complete bypass. When the body temperature dropped to 34°C, the ascending aorta was clamped. For mitral valve replacement, the left atrial was opened. A draw hook was used to accending the anterior wall of the left atrial through a 0.5-cm incision (incision 3). The mitral valve was then visualized. For aortic valve replacement, the aortic root was selected. The lesion valve was excised, and the valve replacement was performed using intermittent sutures. A chest tube was placed *via* incision 1 for closed-chest drainage.

During CPB, goal-directed perfusion was implemented to maintain an oxygen delivery (DO2) level above the identified critical value of 272 mL/min/m^2^ (9), Blood gas analysis was performed every half hour, oxygen delivery (DO2) was calculated using the following equation: DO2 (mL/min/m^2^) = 10 × pump flow (L/min/m^2^) × arterial oxygen content (mL/100 mL), where arterial oxygen content was calculated as follows: Arterial oxygen content (mL/100 mL) = Hb (mg/dL) × 1.34 × Hb saturation (%) + 0.003 × O2 tension (mmHg). (10) Close attention was paid to the hematocrit, which was sustained at around 25%. If the hematocrit went below 25%, perfusion flow or ultrafiltration was utilized to adjust the perfusion rate. Red blood cells (RBCs) were transfused if the hematocrit stayed below 23%.

### Ventilator Setting, Blood Gas Analyses, and Extubation Criteria

Mechanical ventilation was initiated immediately after the induction of anesthesia. The volume-control mode with the following settings was used: tidal volume, 6–10 mL/kg; respiration rate, 8–12/min; and FiO2, 0.4–0.6. Ventilator settings were changed to maintain PaO_2_ at ≥100 mmHg, partial CO2 pressure (PaCO_2_) at 35–45 mmHg, and pH at 7.35–7.45. During CPB, tidal volume was reduced to a small tidal volume (1–1.5 mL/kg). After CPB, the tidal volume and respiration rate were the same as described above after full reexpansion of the lungs by several manual hyperinflations (peak pressure at 20–30 mmHg), and thorough intratracheal suctions.

Blood gas analysis (BGA) was measured before surgery and within 30 min of each time point. Pulmonary function parameters were calculated as follows: PFR = PaO2/FiO2.

Assessable metrics were selected through the collaboration between anesthesiologists and cardiac surgeons, which included assessment of postoperative bleeding, stability of hemodynamic parameters, vasopressor requirement and adequacy of gas exchange. Remifentanil and propofol would be withdrawn if these requirements were met before closing the chest wall.

Patients were extubated when they met the following extubation criteria: (1) hemodynamic stability with reasonable urine output and warm peripheral extremities; (2) PaO_2_ ≥ 75 mmHg and PaCO_2_ ≤ 50 mmHg with adequate spontaneous respiration under FiO_2_ ≤ 0.4 and end of expiratory pressure ≤5 cm H_2_O; (3) awake and able to respond to commands without new neurological symptoms; (4) no active bleeding with a reasonable change in hemoglobin and no requirement for volume replacement; and (5) no reasonable fear of reintubation.

### Data Collection

Trained staff collected detailed data, including the clinical cost for each patient, from the electronic medical records at our medical center. The baseline characteristics collected for each patient involved age, gender, height, weight, body mass index (BMI, calculated based on height and weight recorded by the nurse on the day of hospital admission), diabetes mellitus, hypertension, chronic obstructive pulmonary disease (COPD), left ventricular ejection fraction (LVEF), preoperative hemoglobin, hematocrit (Hct), preoperative serum creatinine (sCr); eGFR (estimated glomerular filtration rate, calculated based on the Modification of Diet in Renal Disease formula).

BGA data before surgery and the end of surgery were also recorded. BGA was measured within 30 min of each time point. Pulmonary function parameters were calculated as follows: PFR = PaO_2_/FiO_2_, alveolar-arterial oxygen gradient (AaDO_2_) = (760−47) × FiO_2_−PaCO_2_/0.8−PaO_2_.

Intraoperative data: type and duration of surgery, CPB time, aortic cross-clamp time, plasma and red blood cell infusion, dosage of sufentanil, remifentanil and rocuronium bromide.

Postoperative data: LVEF, hemoglobin, hematocrit, sCr, eGFR, extubation time, length of ICU, postoperative hospital stay.

Postoperative adverse events during the hospital stay included death, heart failure requiring intravenous medical therapy (e.g., inotropes, furosemide) beyond the fourth postoperative day, myocardial infarction, symptomatic stroke, and acute renal failure requiring hemofiltration. Assessment of delirium was performed preoperatively (baseline) and postoperatively at 12-h intervals or as needed according to the ICU patient’s condition using the confusion assessment method (CAM). When patients were discharged from the ICU to the surgical floor, delirium was also assessed using CAM.

### Statistical Analyses

All statistical analyses were performed using the SPSS statistical package, version 25.0 (SPSS Inc., Chicago, IL, USA). Data are expressed as mean ± standard deviation. Differences between groups were evaluated using the Mann–Whitney U test for continuous variables and Fisher’s exact test for binary categorical variables. Univariate and multivariate logistic regression analyses were performed to identify predictors of prolonged delayed extubation. Receiver operating characteristic (ROC) curves evaluated the sensitivity and specificity of predicting successful on-table extubation. Frequencies and percentages were used for categorical variables. A *p*-value of <0.05 was considered statistically significant.

## Result

We successfully extubated 71 (76.3%) patients within 1 h after surgery using the on-table (in operation room) extubation strategy and no patients received any reintubation, 22 patients did not meet the extubation criteria within one hour after surgery; thus, delayed extubation was performed in the intensive care unit (ICU). When the 71 on-table extubated (OTE) patients were compared to the 22 delayed extubation (DE) patients, the OTE group’s general characteristics did not differ substantially from those of the DE group (Table 1).

**Table 1:** Demonstrates a comprehensive list of preoperative characteristics of the participants.

	OTE group	DE group	*p*-value
Number of patients	71	22	
Age (years)	72.11 ± 3.16	73.27 ± 2.83	0.127
Body mass index (kg/m^2^)	22.39 ± 3.26	23.13 ± 3.25	0.355
Male gender, *n* (%)	34(47.9)	10(45.5)	0.842
Hypertension, *n* (%)	23(32.4%)	10(45.5%)	0.263
Diabetes mellitus, *n* (%)	10(14.1)	1(4.5)	0.660
Previous stroke, *n* (%)	3(4.2%)	1(4.5%)	1.000
Atrial fibrillation, *n* (%)	39(54.9%)	12(54.5%)	0.809
Coronary artery disease, *n* (%)	14(19.7%)	6(27.3)	0.646
Current smoker, *n* (%)	7(9.9%)	3(13.6%)	0.916
Euroscore II	3.94 ± 0.82	3.86 ± 0.73	0.661
NYHA CLASS			
I	11	2	0.722
II	50	17	
III	10	3	
End-diastolic length of the left ventricle (mm)	53.04 ± 9.31	52.14 ± 7.82	0.680
End-systolic length of the left atrium (mm)	51.72 ± 10.62	49.45 ± 8.16	0.361
Preoperative LVEF (%)	61.66 ± 7.36	61.36 ± 6.76	0.866
Pulmonary hypertension, *n* (%)	40(56.3%)	11(50.0%)	0.602
Preoperative hemoglobin (g/L)	129.21 ± 16.31	134.27 ± 18.75	0.223
Preoperative Hct (%)	39.26 ± 8.00	39.71 ± 5.31	0.805
Preoperative sCr (μmol/l)	79.87 ± 15.42	84.61 ± 12.95	0.195
Preoperative eGFR (mL/min·1.73 m^2^)	83.69 ± 21.35	81.76 ± 16.96	0.705
Preoperative PaO_2_/FiO_2_	387.68 ± 32.48	379.71 ± 11.53	0.274
Preoperative PaCO_2_	40.82 ± 3.05	40.58 ± 2.90	0.748
Preoperative PA-ao_2_	16.44 ± 5.14	17.51 ± 5.22	0.408
Lactic acid	1.31 ± 0.42	1.33 ± 0.39	0.851

*Percentages are given as total within group; Continuous data are expressed as mean ± standard deviation.*

*eGFR, estimated glomerular filtration rate (calculated using the Modification of Diet in Renal Disease (MDRD) formula); sCr, serum creatinine; Hct, hematocrit; LVEF, left ventricular ejection fraction; NYHA, New York Heart Association.*

### Intro-Operative and Post-Operative Data

Patients in the DE group underwent longer surgery time, longer aortic occlusion clamping time, longer cardiopulmonary bypassing time than patients in the OTE group, and significant decreases in PFR (PaO2/FiO2) were observed at the end of surgery in the DE group (*p* < 0.001). The dose of sufentanil, rocuronium and remifentanil was higher in the DE group, and this change was associated with the prolonged operation time in the DE group. Patients in the OTE group had shorter postoperative mechanical ventilation time, shorter intensive care unit time, and shorter postoperative hospital length of stay (*p* < 0.05) (Table 2).

**Table 2:** Intraoperative parameters and postoperative parameters.

	OTE group	DE group	*p*-value
Number of patients	71	22	
Operation type			
Mitral valve surgery	52	13	0.206
Aortic valve surgery	19	9	
Operation time (min)	217.48 ± 27.83	275.91 ± 77.22	0.002
Aortic occlusion clamping time	76.49 ± 16.00	126.55 ± 54.85	0.001
Cardiopulmonary bypass time (time)	112.87 ± 18.91	160.77 ± 52.17	0.001
Red cell transfusions, *n* (%)	8(11.3%)	5(22.7)	0.049
Plasma transfusions, *n* (%)	4 (5.6%)	2 (9.1%)	0.883
Fluid balance (mL)	1228.87 ± 268.42	1277.27 ± 209.10	0.382
Urine output	601.97 ± 318.80	620.45 ± 343.53	0.816
Sufentanil	51.53 ± 6.37	57.86 ± 8.00	0.002
Rocuronium	115.15 ± 25.363	134.86 ± 26.552	0.003
Remifentanil	2.38 ± 0.58	2.96 ± 0.74	0.003
Postoperative PaO_2_/FiO_2_	334.36 ± 54.01	200.14 ± 44.325	0.001
Postoperative PaCO_2_	40.19 ± 4.00	38.86 ± 4.66	0.200
Postoperative lactic acid	1.23 ± 0.37	1.31 ± 0.28	0.361
Postoperative mechanical ventilation time^a^ (min)	22.63 ± 4.89	425.29 ± 155.33	0.001
Intensive care unit time (h)	15.44 ± 3.40	23.65 ± 9.50	0.001
Postoperative hospital stay (d)	7.90 ± 1.43	11.33 ± 1.56	0.001
First Postoperative day drainage (mL)	183.94 ± 57.26	206.19 ± 60.79	0.126
Second Postoperative day drainage (mL)	60.49 ± 19.55	59.52 ± 15.96	0.836

^a^ *The time from the end of operation to extubation.*

**Figure 1:**
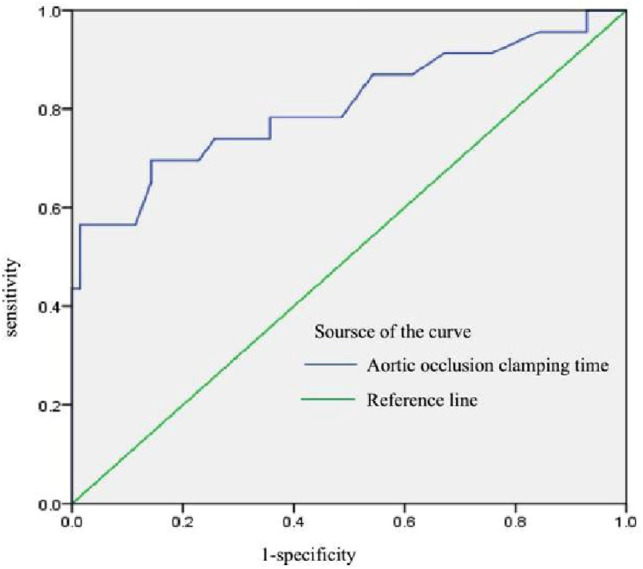
Receiver operating characteristic curve for aortic occlusion clamping time.

As our data showed that operative time, aortic occlusion clamping time, cardiopulmonary bypassing time and the dose of sufentanil, rocuronium and remifentanil were associated with on-table extubation, and significant decreases in PFR (PaO_2_/FiO_2_) were observed at the end of surgery in DE group(*p* < 0.001). Receiver operating characteristic (ROC) curves were used to evaluate the sensitivity and specificity of these variables regarding on-table extubation feasibility. Aortic occlusion clamping time was the most significant predictor of successful on-table extubation, with an AUC of 0.81 (*p* < 0.01) (Figure. 1). Notably, using a cut-off of 92 min, aortic occlusion clamping time was able to predict the success of OTE with a sensitivity of 69.56% and a specificity of 85.71%.

### Postoperative Adverse Events

The rate of acute kidney injury (AKI) was higher in the DE group (2.82%, 27.27%; *p* = 0.001). In contrast, there was no significant difference between delirium, arrhythmia, and thoracentesis incidence.

### months Follow-up Data

There were no prosthetic valve-related complications or death reported during the follow-up period. In addition, there were no significant differences between the two groups in left ventricular end-diastolic diameter, left atrial diameter, LVEF, pulmonary arterial pressure, or heart function after the operation, which illustrated that there was no significant difference in efficacy between the two groups (Table 3).

**Table 3:** 3 months follow-up data comparison between two groups.

	OTE group	DE group	*p*-value
End-diastolic length of the left ventricle (mm)	49.75 ± 7.60	51.43 ± 7.74	0.377
End-systolic length of the left atrium (mm)	47.96 ± 10.33	43.81 ± 7.49	0.773
LVEF (%)	57.47 ± 9.37	56.81 ± 8.76	0.090
Pulmonary artery systolic pressure	34.50 ± 4.79	5.78 ± 4.76	0.483
Perivalvular leakage	0	0	

### Cost Analysis of Different Extubation Treatment

As illustrated in Table 4, in both groups, the total cost and medication cost of the DE group were higher than that of the OTE group (*p* < 0.001), while the surgery cost was similar between the two groups.

**Table 4:** Cost analysis of different extubation treatments.

	OTE group	DE group	*p*-value
Total cost (yuan)	105809.76 ± 11519.21	124094.27 ± 15576.02	0.001
Medication cost (yuan)	10248.93 ± 1617.34	13940.99 ± 2257.97	0.001
Non-medication cost (yuan)	95560.83 ± 10970.79	110153.28 ± 15061.67	0.001
Surgery cost (yuan)	15327.44 ± 4407.39	15035.90 ± 2131.13	0.677
Other medical cost (yuan)	80233.39 ± 11924.29	95117.38 ± 15075.72	0.001

## Discussion

Generally, complex valve surgery necessitates a more prolonged CPB, resulting in an extended duration of mechanical ventilation and ICU stay (11, 12). Delayed extubation is primarily reserved for high-risk individuals at high risk of serious cardiorespiratory complications. As a new recovery concept, early extubation of patients from mechanical ventilation within a few hours after heart surgery may minimize the length of ICU and hospital stays and enhance clinical outcomes (13). Given that the practicality of early extubation has remained limited to elective patients, it is crucial to investigate the predictive factors associated with the successful implementation of this procedure.

Our retrospective analysis demonstrated that on-table extubation is safe and feasible in people aged above 60 years undergoing mitral or aortic valve surgery *via* thoracic right-anterior minimal incision combined with serratus anterior plane block (SAPB). The majority (76%) of older patients could be successfully extubated in the operating room at the end of the surgery, resulting in better clinical outcomes if they meet the extubation criteria. The OTE rate of our study was lower than Ziae Totonchi’s study, whose OTE rate was 96% since the patients were younger (49.26 ± 13.26 years), most surgeries performed (83.3%) were coronary artery bypass grafting, and the CPB time was shorter (57.04 ± 23.05 min) (14). In contrast, the OTE rate of our study was higher than a previous study in pediatric patients after congenital cardiac surgery (53.1%) (15). Patients in the OTE group had shorter postoperative mechanical ventilation time, shorter intensive care unit time, and shorter postoperative hospital length of stay (*p* < 0.05). The rate of acute kidney injury (AKI) was higher in the DE group (2.82%, 27.27%; *p* = 0.001). The total cost and medication cost of the DE group were higher than that of the OTE group (*p* < 0.001). Based on our findings, we concluded that if patients met the OTE criteria, it could be a more cost-effective method to achieve a better clinical outcome.

Prolonged operative time, aortic occlusion clamping time, and cardiopulmonary bypassing time were inversely associated with the ability to accomplish OTE. In elderly patients, the incidence of postoperative complications and perioperative mortality is significantly increased when the CPB time is prolonged. The study showed that the longer the CPB time, the higher the perioperative mortality. Compared to patients with a CPB time shorter than 75 min, the perioperative risk of death with a CPB time longer than 75 min increased sharply (16). Impaired pulmonary gas exchange is a common complication after cardiopulmonary bypass (CPB) (6). In our study, the mean CPB time in the OTE group was 112.87 ± 18.91 min, compared to 160.77 ± 52.17 min in the DE group. Aortic occlusion clamping time was the most significant predictor of successful on-table extubation with an AUC of 0.81 (*p* < 0.01).

The dose of sufentanil, rocuronium and remifentanil was higher in the DE group, and this change corresponds to the prolonged operation time in the DE group. We believe that the anesthetic technique plays an important role too. Herein, the anesthetic protocol focused on minimizing sedating drugs, encouraging shorter-acting intraoperative opiates, and performing SAPB to decrease the sufentanil dose during operation (17). SAPB with general anesthesia (GA) can provide high-quality analgesia and quick rehabilitation (18). During CPB, mechanical ventilation was switched to a small tidal volume (1–1.5 mL/kg, the FiO_2_ was 50%), which not only does not affect the surgical procedure but may also reduce pulmonary injury during CPB, particularly in valve surgery *via* thoracic right-anterior minimal incision (19). The need for postoperative analgesia during the recovery area stay was considerably higher in the remifentanil group than in the sufentanil group (20). In our hosptal, we used sufentanil combined with remifentanil.

Nevertheless, there are some limitations to this study. This was a single-center retrospective study with a small sample size. A prospective, multicenter, randomized controlled trial is warranted in the future to support our result. Moreover, the follow-up time of our study was short, and a longer follow-up is needed to identify the long-term outcome of on-table extubation. In this retrospective study, we implemented on-table extubation under our present anesthesia protocol, but more studies are needed to determine the outcome of OTE under different anesthesia conditions.

## Conclusions

On-table extubation after mitral or aortic valve cardiac surgery is a relatively new strategy. Herein, we performed OTE in accordance with our enrollment criteria and our hospital’s anesthesia protocol. Importantly, we uncovered that successful on-table extubation in the operative room was associated with a better clinical outcome and high cost-effectiveness.

## Data Availability

The original contributions presented in the study are included in the article/Supplementary Material, further inquiries can be directed to the corresponding author/s.
